# Editorials

**Published:** 1890-12

**Authors:** 


### HOMŒOPATHIC PHYSICIAN,

A MONTHLY JOURNAL OF

HOMŒOPATHIC MATERIA MEDICA AND CLINICAL MEDICINE.

"If our school ever gives up the strict inductive method of Hahnemann, we are lost, and deserve only to be mentioned as a caricature in the history of medicine."—CONSTANTINE HERING.

Vol. X. DECEMBER, 1890. No. 12.

### EDITORIAL.

THE DOSE.—We call the attention of our readers to Dr. Lilienthal's article entitled "Another Dose about the Dose," in this number of THE HOMŒOPATHIC PHYSICIAN.

There are many members of the homœopathic school who are asking the same question; why is not the dose of the remedy indicated in all works on treatment?

Dr. Lilienthal has given an answer, and has happily illustrated it by cases. In a private letter to this journal, the venerable Doctor declares his inability to solve the question, and, with charming frankness, declares himself "a mongrel" who could never be admitted to the International Hahnemannian Association or the Legion of Honor of the homœopathic profession.

He appeals to us to aid him in his answer. We are as helpless as himself for a solution of the question. Only time, by the ponderous and exceedingly slow process of evolution, can bring an answer. Homœopathic physicians can help that evolution by strictly and faithfully applying the law of the similars, giving only one drug at a time, and seeking only the smallest dose that will cure. This will be recognized as the old and oft-repeated advice of Hahnemann, and that of all the old guard of his faithful disciples.

Those who follow such advice are, however, derisively called "symptom coverers" by the majority who, nominally of our school, yet feed from the pathological flesh-pots. This stigma is, of course, unjust. The real symptom coverer is the surgeon who cuts out the ovaries to cure painful menstruation ; the doctor who relieves an "overloaded bowel" by a purgative, or deadens the sensibility to pain by an opiate, or applies a fly-blister for an inflammation. Those who do such things are the *real* symptom coverers.

On the other hand, the physician who takes the totality of the symptoms is seeking to gain a complete knowledge of the whole condition, to get a perfect picture of the disease. In applying his remedy he simply ensures that the remedy, the messenger, the agent which he sends, shall actually reach the spot where the enemy is located, and, coming in collision with him, shall annihilate him. Now, then, the dose must be so graduated that this triumph over the disease shall be just sufficient to accomplish the end sought, and yet not increase the suffering and danger to the system that must endure the shock of the battle. What dose shall this be? It is impossible to say. We must simply give the remedy according to our light, and, watching carefully the result, learn enough to advance us from one potency to another, until we have arrived at a conclusion. This method of procedure is strictly scientific. It has the true character of the scientific investigation of the physicist and chemist. When the physicist, the chemist, or the electrician seeks to solve some scientific question, he has recourse to a laboratory and to scientific instruments. The attempt with the apparatus, under such circumstances, to find a solution to his question is called an experiment. Prof. Tyndall happily called it "addressing a question to nature." Every phenomenon in nature is influenced by so many factors that means must be taken to shut out all of these factors save the particular one we are dealing with. This suggests apparatus. As every factor except the one under consideration interferes with the solution of the question, invention is taxed to devise means for shutting out these other factors. Thus modifications in the apparatus are made, and so it grows into the many singular forms and complicated devices that distinguish scientific appliances. The plan of the apparatus is such, that, as far as possible, only *one* influence shall be present, in the experiment, at a time to produce the effect to be studied.

This is well illustrated in Prof. Tyndall's striking and beautiful investigations upon the Diathermancy of Air; the diamagnetic polarity of miscellaneous bodies generally supposed to be insusceptible to magnetism, and his experiments on magne-crystallic action.

Applying this method to the question of a prescription, we find that, if we would form any conclusion as to the value of a medicine in any given case of sickness, we must give it *alone.* No other factor, no "adjuvant means" of any kind whatsoever must be allowed to obscure our judgment and prevent an indisputable conclusion as to the efficacy of the medicine. Only one potency can be given at a time, and a sufficient interval must be allowed for us to be able to note its results and see our way clear to the next step in the progress of the patient.

From this point of view we cannot see how any physician can claim to have any clear understanding of what he is seeking to do with a patient when he alternates remedies, alternates potencies, or uses adjuvant means of treatment. With such prescribing, the question of medical treatment, the question of the dose will never be settled, and patients will continue to endure prolonged sufferings and uncertainty as to cure.

The plain road, therefore, toward that certainty in medicine which characterizes astronomy, and *which*—say what the skeptics will—is *attainable,* is by the similar remedy, the single remedy, and the minimum dose exactly as indicated by Hahnemann.

The continuation of that plain road is to be sought in *the proving of the potencies.* The symptoms of crude drugs are as different from the symptoms produced by the potencies as the symptoms of one drug differ from those of another. All these varieties of effect of the potencies of the same drug should be carefully separated, and, in any case of sickness, only that potency given whose symptoms most nearly correspond with the sick condition. Then permitting the dose given to exhaust its action before giving another one, or changing the remedy, will clear the way for an intelligent conclusion as to the scope of any remedy or of any potency. An immense number of such observations made by a great number of intelligent physicians and recorded in the journals will enable some Kepler in the homœopathic school to formulate a law for the selection of the potency. The *repetition* of the dose is, however, a somewhat simpler matter. Any homœopathic physician who has ever given a remedy that was indisputably the simillimum, knows well the gratifying and prompt response he gets in the improvement of the patient's condition. He can trace with surprising clearness the decline of the painful or dangerous symptoms from day to day or from hour to hour. He knows well the folly of repeating the dose whilst this improvement continues. He perceives most clearly when the moment has arrived in which the dose has exhausted its action and it is time to administer another one. The question, therefore, is settled for him.

On the other hand, he who makes random or careless prescriptions, gives medicines in alternation, and in crude doses never witnesses any of these phenomena. For him they are a sealed book. Hence his uncertainty, his open scepticism, his hostility to the very principle he claims to espouse, and so arises a divergency of views which is an exact measurement of the angle between the satisfactory experience of the careful student of symptomatology, and the negative experience of the superficial or eclectic prescriber. W. M. J.

---

GRAFTS.—In our last number Dr. Yingling calls attention to an article in the September *Homœopathic Recorder*, and he asks for experience in the use of grafts.

Even slight observation will show the *Recorder's* article is written from the commercial standpoint and is not consonant with facts. "There is or was," says A. J. T., in the article in the *Recorder*, "still another way of making cheap sets of high potencies. Eight or ten students would club together and buy an original set of liquid high potencies from a pharmacist, then subdivide them into as many sets of vials filled with alcohol." Then an attempt is made to show that these latter are of no value.

Now, if the "original" potencies were of any value, we should like A. J. T. to show us why the next higher number made from these proved valueless.

When such men as Hering, Lippe, Fellger, Bayard, Wells, and many others successfully used grafts, we shall ask for higher authority before we cease using them, as we have been doing for eighteen years.

Dr. Hering once told us of a severe case of pleurisy he was called to treat, in which the pain was agonizing. After determining the remedy he was chagrined to find the vial empty. It being late at night and some distance from his office, he was momentarily at a standstill. This lasted but a moment. He filled the empty vial with Sac-lac., succussed it thoroughly, administered it, and had a gratifying result.

Grafts when properly made are of value. Quantity has no place in homœopathics: quality and law are the essentials.

G. H. C.

---

Dr. Dudgeon on High Potencies.—Attention also is called to another article in the *Recorder*, extracted from *The Homœopathic World* for August. In this Dr. Dudgeon (to whom we all owe thanks for his translations of many of Hahnemann's works) tries to show that those who use the potencies of Fincke, Skinner, Lehmann, Jenichen and others are not following the teachings of Hahnemann, and, therefore, are not entitled to the name of Hahnemannian. Indeed, Dr. D. claims that the use of such potencies "is directly opposed to his (Hahnemann's) teaching and practice." We are aware that some of these potencies are made in a manner different from that recommended by Hahnemann; and we also know that men of reliance have put them to actual test and have found them beneficial. Further, the potency has nothing whatever to do with Hahnemann's Homœopathy, and Dr. D. knows it; the law is the all-requisite—and its corollaries. We are under the impression that a stereotyped argument of the opponents of high potencies is that the followers of Hahnemann have made no advance since his day, and that they are not progressive. The results of a strict adherence to Hahnemann's teachings, and the use of these potencies are sufficiently well known; and this, in spite of many flings, is, after all, the only argument needed.

Says Dr. Dudgeon: "Those of us who practice Hahnemann's system *with modifications* (we italicize) suggested by experience and reflection, which we imagine, perhaps mistakenly, to be improvements [improvements on a law of nature? G. H. C.] do not announce ourselves as Hahnemannians or bestow on those who differ from us uncomplimentary epithets." And you do right in not announcing yourselves as Hahnemannians, but you do wrong, and are a fiction, when you take the name of homœopathist.

What is usual with those "who practice Hahnemann's system with modifications"? In our experience that simply means Morphia for pains, Quinine for intermittents, and the use of other hurtful drugs. We know of numerous instances in which we have been convinced that through the use of these crude drugs, these men "who practice Hahnemann's system with modifications" have caused the death of patients. And this was done under the name of Homœopathy.

Are the honest followers of Hahnemann to stand idly by and say nothing of the harm that is being done by these men, and thus impliedly indorse them as homœopathists?

For those whose practice is based upon the law of similars, and who adhere to that law, whether they use the crude tincture or the CM, we have only praise. But what of those who are constantly doing all in their power to belittle Homœopathy, by resorting, in many cases, to treatment that would put to the blush an old-school drugger? What of those of whom the allopathists say, "They are living a lie"?

We should feel recreant to our trust if we ever failed to denounce such treatment. We have a mission. Its proper execution demands that we should teach the people the difference between the genuine and the false; the right and the wrong; fact and fiction; law and empiricism; the curative and the palliative; and endeavor to have them know that temporary expedients have no rightful place in the treatment of the sick; and that the only right and safe treatment is that based on Nature's law: SIMILIA SIMILBUS CURANTUR. G. H. C.

---

THE following, in substance, is related of Benjamin Franklin: On being asked if he thought a pail filled to the brim with water would overflow if a fish were placed in it, he replied, Gentlemen, when in doubt in respect of anything that can be put to a test, I always make the test before giving a decision.

Dr. Yingling has, perhaps unconsciously, followed Franklin's advice. We commend the same to all who rail against high potencies. G. H. C.

---

THE OTHER SIDE.—Upon page 548 of this number Dr. T. F. Allen asks for the other side of the question of suppressing gonorrhœa by injections. Dr. Allen, however, does not call it suppression, but nothing else can be made of the treatment he advises. The other side is the side which decries the suppression of any external disease manifestation, and simply because that side has the best interests of the patient at heart, and knows what harm may result from suppression.

This is a truism: a sycotic gonorrhœa made to disappear by the use of injections, converts what might be terminated favorably to the patient into a latent, deep-seated affection which gives rise to a host of symptoms of a character that may simply be defined as infernal. The mental symptoms, particularly, are such as to make the victim of suppression such a burden to himself that he is constantly thinking of suicide. Every Hahnemannian should know this, even though his experience be limited to but one case of sycosis, as that affection was described by Hahnemann, Autenrieth, Bœnninghausen, and Granvogl.

That sycosis does not follow all cases of suppressed gonorrhœa is due to the fact that a large number of such are but simple urethritis, and in these cases astringent injections do little else than cause stricture.

To the victim this is formidable enough, but this is as nothing compared to the condition brought about by suppressing sycotic gonorrhœa. In this state the poor victim has all the *Sheol* he can care for, but not as much of it as should be doled out to him, who, professing a knowledge of beneficent Homœopathy, will resort to means that are not homœopathic.

Dr. Allen asks what is the harm of washing out the discharge from the urethra? We say plainly there is harm. It is far better for the patient to have the discharge continue, and if we could not have him view it from our standpoint we should permit him to do as he pleased, but we should not do anything to cause him suffering which might be life-long. (Unfortunately for the victims of suppression, their lives are apt to be too long for them.)

If Dr. Allen has had any experience in the treatment of sycosis—and he certainly must have had if he knows anything of the teachings of Hahnemann—then he should know of the effects of the treatment he advises. Perhaps his (Dr. Allen's) experience in the treatment of gonorrhœa may enable him to determine between simple urethritis and sycotic gonorrhœa. If he can diagnose without a doubt between the two we wish he would impart that information, for we have never met one who possessed that power.

In the absence of his ability to discriminate between the two affections he should certainly, before advising any treatment, endeavor to stand by the colors he professes to uphold, and, then, when the time arrives for treating cases of gonorrhœa he will be able to do the best possible for his patients by following the teachings of Hahnemann. G. H. C.

---

At p. 398, Vol. VIII, HOMŒOPATHIC PHYSICIAN, will be found an article on "Some Symptoms of Sycosis," to which we respectfully refer Dr. Allen and those who agree with him.

G. H. C.

---

DR. SAMUEL LILIENTHAL.—On Wednesday, November 5th, Dr. Samuel Lilienthal attained his seventy-fifth birthday. Dr.

Lilienthal is widely known by all who are members of the homœopathic school for his extensive and remarkable contributions to the literature of the school. There are few journals of any influence among homœopathists that have not at some time published his contributions. Much of his work has been in the way of translation, much of it entirely original.

He is a *liberal* homœopathist, but openly calls himself a "*mongrel.*" He is the author of an excellent book entitled *Homœopathic Therapeutics*, now in its third edition, in which we fail to find any evidences of mongrelism. On the contrary, under the different diseases, which are arranged in alphabetical order, we find only the most strikingly characteristic indications for the various remedies.

Notwithstanding his advanced age, Dr. Lilienthal still continues his usefulness; the before-mentioned book having been completed only within a few months.

We send him our warmest congratulations, and wish him a continuation of life graced with the honors willingly accorded him by his brethren in the profession. W. M. J.

---

THE TEACHING OF PURE HOMŒOPATHY IN THE COLLEGES.—Our editorial upon this subject in the October number at page 437, brought out a protest which was noticed in the November number at page 483.

We have now another protest, this time from Dr. W. E. Leonard, Professor in the University of Minnesota.

He writes that he has been teaching *The Organon* for four years as a part of the regular course of lectures. He sends us a syllabus of his lectures, from which it appears that in the time from October 9th to May 18th, he gives seven lectures upon *The Organon*, the last being a review lecture.

We are very glad to be able to make this announcement, and add one more to the list of colleges where strict Homœopathy is taught.

At this rate we ought to be able to publish a "Roll of Honor" of such colleges as engage to teach absolutely pure Homœopathy unmixed with the rational therapeutics of the old school. W. M. J.

